# Preclinical Evaluation of the Anticoagulant Effect of an Aqueous Extract of Snow Mountain Garlic

**DOI:** 10.3390/medicina61030429

**Published:** 2025-02-28

**Authors:** Isabel Clark-Montoya, Rosa del Carmen Milán-Segovia, Obed Lemus-Rojero, Osmar Antonio Jaramillo-Morales, Bertha Júarez-Flores, Yolanda Terán-Figueroa

**Affiliations:** 1 Faculty of Chemical Sciences, Autonomous University of San Luis Potosi, Dr. Manuel Nava Martínez Avenue #6, University Zone, San Luis Potosi 78210, Mexico; a201618@alumnos.uaslp.mx (I.C.-M.); milanros@uaslp.mx (R.d.C.M.-S.); 2 Histology and Histopathology Laboratory, Academic Unit of Dentistry, Autonomous University of Zacatecas, Zacatecas 98160, Mexico; obed.lemus@uaz.edu.mx; 3 Division of Life Sciences, Nursing and Obstetrics Department, Campus Irapuato-Salamanca, Guanajuato University, Guanajuato 36612, Mexico; oa.jaramillo@ugto.mx; 4 Desert Areas Research Institute, Autonomous University of San Luis Potosi, San Luis Potosi 78290, Mexico; berthajf@uaslp.mx; 5 Faculty of Nursing and Nutrition, Autonomous University of San Luis Potosi, Niño Artillero Avenue #130, University Zone, San Luis Potosi 78240, Mexico

**Keywords:** Snow mountain garlic, *in vivo*, anticoagulant, chronic toxicity

## Abstract

*Background/Objectives:* In the genus *Allium*, it has been shown that some organosulfate compounds of common garlic and onion have an antiplatelet effect. Still, little is known about the anticoagulant activity of the species, *Allium ampeloprasum* L., specifically Snow mountain garlic; due to its growth site at 6000 m above sea level at temperatures of −10 °C, it could contain different organosulfate compounds. *Methods:* This study evaluated the anticoagulant effect of a lyophilized aqueous extract of S. mountain garlic *in vivo,* toxicity in male Wistar rats for 90 days, and the organosulfate compounds present. *Results:* There was a significant increase (*p* < 0.05) in the area under the curve for bleeding time at doses of 500 and 1000 mg/kg/d of lyophilized aqueous extract of Snow mountain garlic and 100 mg/kg/d of acetylsalicylic acid. The ED_50_ was 366 ± 2.43 mg/kg. For chronic toxicity *in vivo*, no histopathological alterations were found at doses below 500 mg/kg. On the other hand, organosulfate compounds were found in the lyophilized aqueous extract of S. mountain garlic that had not been documented to have an anticoagulant effect. *Conclusions*: We conclude that S. mountain garlic contains anticoagulant compounds which may be candidates for the treatment of thrombosis.

## 1. Introduction

Thrombosis is the most common cause of death among people suffering from cardiovascular disease. It manifests through vascular occlusion, leading to severe conditions such as myocardial infarctions, cerebrovascular accidents, and venous thromboembolism. This issue presents a global public health problem, causing the deaths of 1–5 people per 1000 [1].

The discovery of agents that lengthen coagulation times began in 1916; in 1918, Howell demonstrated the anticoagulant action of extracts of dog liver, called heparin [2,3]. It was 23 years later that Campbell and colleagues isolated and characterized the hemorrhagic agent 3,3’-methylenebis-(4-hydroxycumarin), known as dicumarol or hydroxycumarin, which served as a basis for developing synthetic anticoagulants. Clinical use of warfarin for humans was approved in 1954 [4].

The search for new anticoagulants to overcome the limitations of vitamin K antagonists (VKAs) has been promoted; they must have several characteristics, such as being administered orally, demonstrating a low risk of bleeding, not requiring constant medical monitoring of coagulation, not needing to adjust the dose, and having a low level of interaction with drugs and food, that reduce the effects of blood-material contact (for example, cannula of the extracorporeal life support) and improve their hemocompatibility [5,6,7].

The development of oral anticoagulants to replace AVK has been slow. Dabigatran, an oral thrombin inhibitor, rivaroxaban, and apixaban, which are oral factor Xa (FXa) inhibitors, are currently available [8]; however, bleeding rates from their use are similar to those of warfarin, depending on the pathologies that cause clot formation. Regardless, comparative efficacy data applied in the clinic are limited [9]; they also present several serious inconveniences depending on the medical or surgical problem that a patient is facing, as well as being susceptible to drug interactions [10]. This leads to the search for natural active ingredients that, at a certain point, offer greater safety in their use. One such option is plant- or food-derived substances.

Thus, traditional medicine is used worldwide because it is accessible and culturally accepted [11]. For millions of people, it is the main and/or only source of healthcare. The World Health Organization (WHO) recognizes that traditional medicine must be incorporated into the health systems of each country [12].

One of the most studied species is *Allium*. For example, some aqueous extracts, ethanolic, methanolic, powders, and oils of *A. sativum* L. have been studied which show anticoagulant activity *in vitro*, *in vivo*, and in humans. The chemical compounds which this property has been attributed to are allicin, trisulphide diallyl, disulfid diallyl, and ajoenes. These sulfurous compounds, according to different authors, have a platelet agonist effect [13,14,15], fibrinolytic activity with a delay of up to 12 h [16] in coagulation time [17,18], prothrombin delay [19,20], platelet thromboxane formation 5 [15], improve plasmin generation via t-PA, antithrombin activity III [20,21], inhibit protein C [20], and decrease the coagulation factors V, VII, and VIII; certain levels of protein C, IX, and X [22] have led to the inhibition of 5-lipoxygenase and cycloxygenase through a decrease in thrombocytic aggregation [23]. Similarly, studies of onion (*Allium cepa*) have documented that sulfur compounds and some phenolic compounds also act as antiplatelet agents [24].

However, findings are not yet conclusive, and they depend on the sample size of the studied population, the dose of the sulfur compounds, their mode of action, and how they are consumed to achieve anticoagulant activity.

Although there are many studies on the anticoagulant effect on plants of the genus *Allium*, little is known about *Allium ampeloprasum* L. (Snow mountain garlic); recently, it has been shown to have a fungicidal effect on candida species as well as antiarthritic, anti-inflammatory, and analgesic effects [25,26,27].

Therefore, it is believed that, due to the high altitude (6000 m above sea level) and low temperatures (−10 °C) of its growth, the medicinal and sulfur content properties of this garlic can increase by up to seven times [28,29]. It should be noted that organosulfate compounds are thought to have healing properties [30,31]. In the work of Mehra et al. (2019), the metabolic pathways of sulfur compounds were studied, finding that S. mountain bulbs express genes involved in the biosynthesis of these bioactive compounds [29]. For this reason, it is important to carry out studies on the anticoagulant properties of S. mountain garlic and to try to identify new molecules with anticoagulant properties.

Thus, this work aimed to evaluate the *in vivo* anticoagulant effect of the aqueous extract of S. mountain garlic, its possible toxicity, and the metabolites with sulfur that might be present.

## 2. Materials and Methods

### 2.1. Plant Materials

S. mountain garlic cloves were obtained from the local market on Reforma Street 405A, 553 Historical Center, 78000, San Luis Potosi, Mexico. Those in good condition, without pest marks, or those that had a brown color were selected. A large quantity was purchased to carry out the study.

### 2.2. Chemicals

The chemicals used were ethanol (CTR scientific, Monterrey, Mexico), helium (Praxair, CDMX, Mexico), and paraformaldehyde (Sigma-Aldrich, St. Louis, MO, USA).

### 2.3. Preparation of Aqueous Extract of S. mountain Garlic (Allium ampeloprasum *L.*)

S. mountain garlic cloves were disinfected with 96% ethanol and peeled. Subsequently, 50 g of the bulbs were weighed, and placed in a clean porcelain mortar to be crushed with a pestle; at the same time, 40 mL of distilled water was added until a homogeneous mixture was obtained. Afterward, it was filtered through filter paper (Whatman No. 0 (San Luis Potosi, Mexico), and the aqueous extract (EA) was obtained and stored at −20 °C.

Subsequently, it was lyophilized in 2.5 L with Labconco FreeZone (Kansas City, Missouri, MO, USA) equipment; this mixture is referred to as the lyophilized aqueous extract (LAE) of S. mountain garlic.

### 2.4. An Animal Model to Evaluate Bleeding Time After the Administration of S. mountain Garlic LAE

The research protocol was approved by the Ethics and Research Committee of the Faculty of Nursing and Nutrition of the UASLP with the following registration number: CEIFE-2021-345. A total of 24 male rats of the strain were used.

Wistar rats with a weight of 250 ± 50 g and an age of 12–14 weeks were obtained from the Regional Biosciences Center of the Autonomous University of San Luis Potosi (UASLP). The animals were kept in the animal management area of the Phytochemistry Laboratory of the Desert Zones Research Institute at the UASLP. The rats were divided into 6 treatments (n = 4) for the daily administration of the LAE with an esophageal cannula for 90 days; they were maintained on a normal diet (LabDiet 5001) of 25 g daily and distilled water ad libitum with a 12 h light–dark cycle. After 90 days, the animals were euthanized with intraperitoneal pentobarbital at doses of 120 to 210 mg/kg. Animal management was based on the indications of the Mexican Official STANDARD NOM-062-ZOO-1999 [32].

### 2.5. Bleeding Time in Rats Treated with LAE of S. mountain Garlic

The *in vivo* bleeding time was based on the methodology of Uchechi et al., 2016, implementing the Duke method [30]. The process consisted of placing the rat in a trap, disinfecting the terminal third of the tail with 70% ethanol, and inserting a sterile needle into the lateral vein. Once the first drop of blood began to flow, the bleeding time began to be counted using a stopwatch. A normal bleeding time is 2–3 min [33]. There were 6 treatments administered (n = 4): a negative control with distilled water, 250, 500, 1000, and 2000 mg/kg/d of the LAE, and a positive control with 100 mg/kg/d of acetylsalicylic acid (ASA). The bleeding period was determined at time 0 (before treatment), 30 min, 1 week, 2 weeks, 4 weeks, 8 weeks, and 12 weeks after treatment with LAE.

### 2.6. Chronic In Vivo Toxicity in Rats Treated with LAE S. mountain Garlic

The male Wistar rats were sacrificed at 12 weeks via an overdose of pentobarbital, as indicated in NOM-0062-ZOO-1999 [32]. After death, the esophagus, stomach, duodenum, liver, and kidney were removed and placed in 10% paraformaldehyde for 24 h. Subsequently, for histological analysis, the tissues were embedded in paraffin (CTR Scientific, Monterey, Mexico), and the samples were cut at 4 microns using a microtome (Micro, Wetzlar, Germany), and stained with Hematoxylin (Jalmek, Mexico) and Eosin (Hycel, Mexico). Histopathological changes including inflammation and lymphoplasmacytic infiltration were observed under a Leica DM500 microscope (Heerbrugg, Switzerland), and the severity of the changes was marked by the pathologist using a score from 0 to 4 (0 = normal, 1 = minimal, 2 = moderate, and 4 = severe).

### 2.7. Identification of Compounds Using GC-QToF-MS

The solid-phase microextraction (SPME) technique was performed to retain volatile and semi-volatile compounds of the sample. The LAE was placed in a 50/30 μm DVB/CAS/PDMS, with a Staflex (2 cm, gray) fiber holder (Supelco, Bellefonte, PA, USA). Then, the identification of LAE compounds using GC-QToF-MS was performed using the gas chromatography technique Q-ToF-MS with a Agilent 7200 gas chromatograph, with a mass detector with time flight tube (Agilent, Santa Clara, CA, USA), a column of DB-WAX (measuring 30 m in length (m), 0.250 mm in diameter (mm), with a 0.25 film (μm) (Agilent, Santa Clara, CA, USA)) and a helium carrier gas at 99.999%. Tests were conducted at a temperature of 50 °C for 4 min, from 50 to 150 °C to 2 °C/min, at 150 °C per 5 min (split/splitless and with an ionization source of EI, 10 microV). Organosulfate compounds were identified using a MassHunter GC/MS (Acquisition B.07.02.1938, 8 September 2014, Copyright ©, 1989–2014, Agilent Technologies, Inc., Hong Kong).

### 2.8. Statistical Analysis

Due to the characteristics of our data, the normality assumptions of parametric tests may not be reliable with small sample sizes (n = 4 per group of treatment), for this reason, we performed a Kruskal–Wallis non-parametric test to assess overall group differences, followed by Dunn’s post hoc test for pairwise comparisons, were applied using Python 3.13.1.

The area under the curve (AUC) has been calculated using the trapezoid method by GraphPad Prism version 8. The comparison of AUC data for the 90-day doses was conducted using a one-way ANOVA, followed by a post hoc Dunnett’s test to compare group means.

The logarithmic dose–response curves of AUC *in vivo* bleeding time were created to determine the effective dose 50 (ED_50_). The doses of each experimental treatment (n = 4) were 0, 250, 500, and 1000 mg/kg/d of LAE.

## 3. Results

### 3.1. Bleeding Time in Rats Treated with LAE of S. mountain Garlic

We evaluated the effect of LAE on bleeding times in rats at different doses (250, 500, 1000, and 2000 mg/kg) compared to a control and a 100 mg/kg ASA.

Significant overall differences in bleeding times across treatment groups were observed at 2 weeks (*p* < 0.05) and 4 weeks (*p* < 0.01). On the other hand, there were significant increases in bleeding times for 500 and 1000 mg/kg treatments at 1 week, 2 weeks, and 4 weeks, with the strongest effects observed at 2 and 4 weeks (*p* < 0.05). Meanwhile, the lack of statistical significance at later time points for the 2000 mg/kg dose suggests a saturation effect at higher concentrations. The 100 mg/kg ASA group demonstrated significant effects at 30 min and 2 weeks, consistent with its known pharmacological profile. These findings indicate that the LAE exerts a dose and time-dependent effect on bleeding times, particularly at intermediate doses. However, from week 8 onwards, the pairwise statistical effects start to attenuate across all groups; nevertheless, the multiple comparison differences remain statistically significant at both 8 and 12 weeks (*p* < 0.01), as indicated by the Kruskal–Wallis test (Table 1). 

### 3.2. The Area Under the Curve (AUC) of the Anticoagulant Effect In Vivo of the LAE S. mountain Garlic

**Figure 1:**
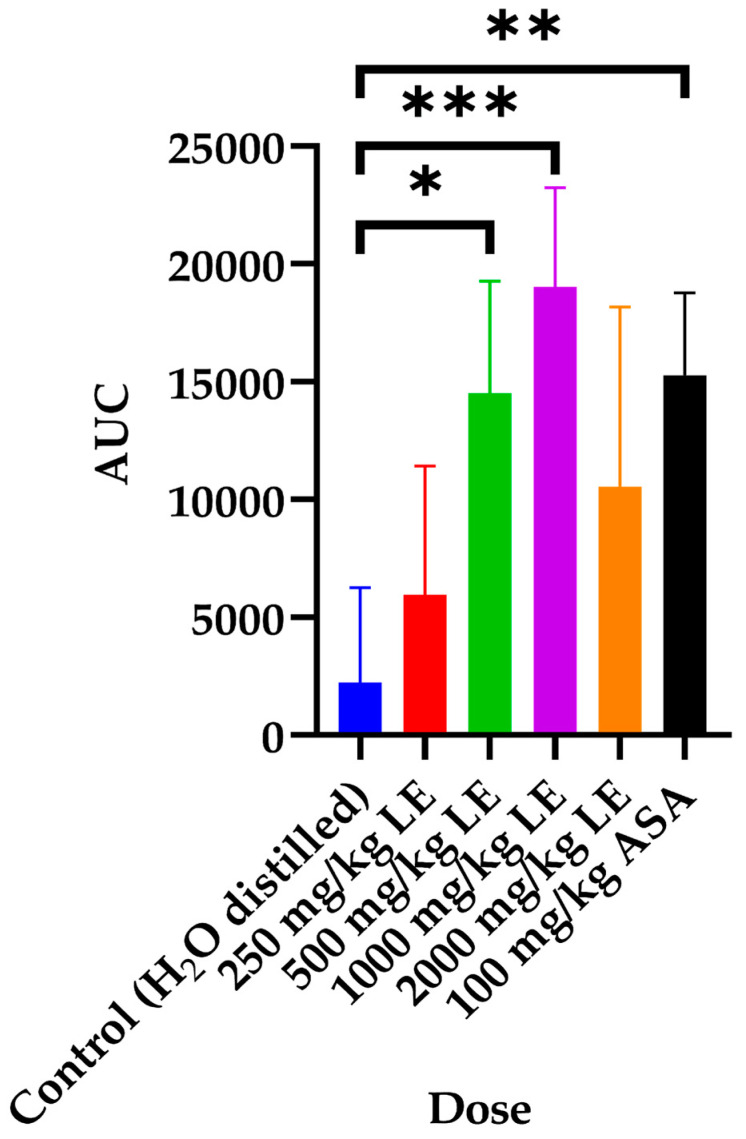
The area under the curve (AUC) 90 days after treatment: control, 250, 500, 1000, and 2000 mg/kg/d of LAE and 100 mg/kg/d ASA. An ANOVA was performed with a post hoc Dunnett to observe the differences between groups. The symbols indicate significant differences compared to control of * (*p* < 0.05), ** (*p* < 0.01), and *** (*p* < 0.001).

In the AUC, a significant increase (*p* < 0.05) was observed compared to the control, with doses of 500 and 1000 mg/kg/d and with the dose of 100 mg/kg ASA, showing an anticoagulant effect superior to that of ASA (see Figure 1).

### 3.3. ED_50_ In Vivo of the Anticoagulant Effect of LAE S. mountain Garlic

**Figure 2:**
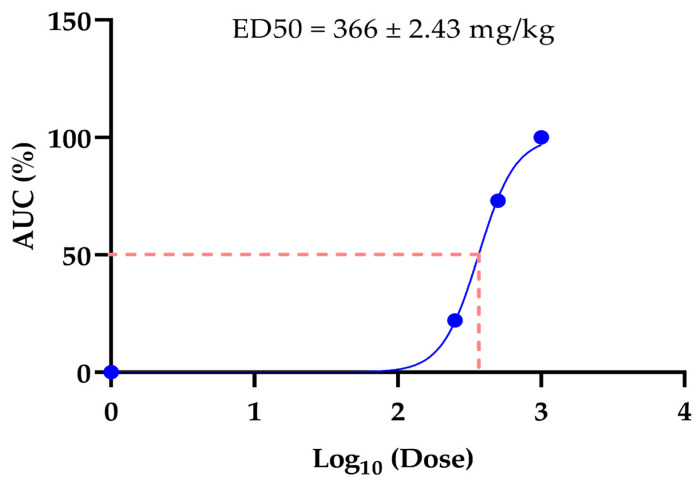
Dose–response curve of AUC *in vivo* of 90 days of treatment of LAE.

The *in vivo* ED_50_ was taken as an AUC, with a low dose of 250 mg/kg up to a high dose of 1000 mg/kg, since it showed significant differences from the control. The ED_50_ was 366 ± 2.43 mg/kg (see Figure 2).

### 3.4. Chronic In Vivo Toxicity in Rats Treated with LAE of S. mountain Garlic

**Figure 3:**
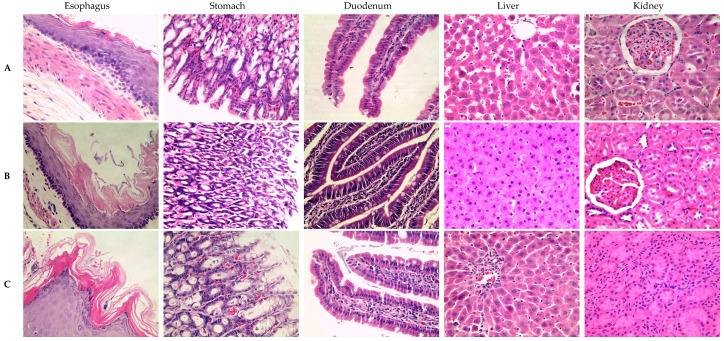

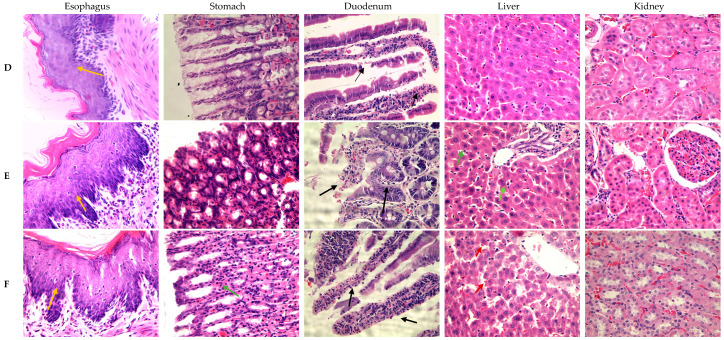
Comparison of the histopathological analysis of 90-day chronic toxicity with the doses and organs studied. The letters (**A**) indicate the control rats, (**B**) the doses of 250 mg/kg/d of LAE, (**C**) the doses of 500 mg/kg/d of LAE, (**D**) the doses of 1000 mg/kg/d of LAE, (**E**) the doses of 2000 mg/kg/d of LAE, and (**F**) the doses of 100 mg/kg/d of ASA. Black arrows indicate the duodenal ulcers; yellow arrows indicate acanthosis in the esophagus; green arrows indicate inflammatory infiltrate in the stomach and liver; red arrows indicate hemorrhage in the liver. A 40× lens was used in the microscope.

The tissues stained with Hematoxylin and Eosin for the histopathological analysis of the tissues of the esophagus, stomach, duodenum, liver, and kidney did not show histopathological findings compared with the negative control at doses of 250 and 500 mg/kg/d; however, at doses of 1000 and 2000 mg/kg and 100 mg/kg of LAE, ASA led to duodenal ulcers and mild severe lymphoplasmacytic infiltrate (see Figure 3). In the tissues, we observe the following events: the erosion of the duodenal mucosa; acanthosis due to epithelial hyperplasia that is a result of the accumulation of cytoplasmic glycogen in the squamous epithelium in the esophagus; inflammation due to lymphoplasmacytic inflammatory infiltrate, which is caused by the presence of lymphocytes in the stomach; and hemorrhage due to the accumulation of hematomas in the liver parenchyma.

### 3.5. GC-QToF-MS Analysis

**Figure 4:**
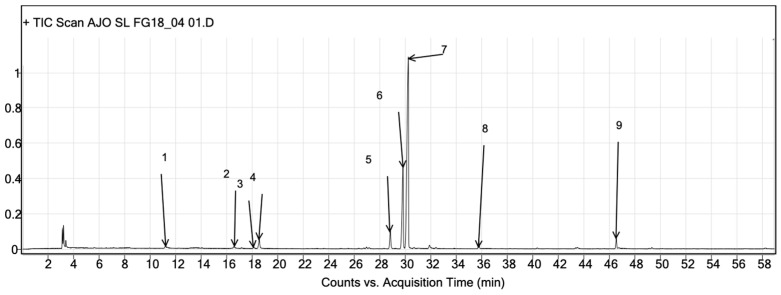
GC-QToF-MS of the organosulfate compounds of S. mountain garlic. The compounds of each peak are described in Table 2.

The genus *Allium* is characterized by an abundance of sulfur compounds. For this reason, we used GC-QToF-MS to identify the organosulfur compounds of LAE from the S. mountain garlic. Nine organosulfur compounds were identified, as shown in Table 2 and Figure 4.

## 4. Discussion

Plants and plant foods have been shown to have promising results in aiding in the treatment of thrombosis [18]. According to the WHO, 80% of the population uses traditional medicine as a primary care option for various diseases [11]. Medicinal plants represent the ancestral wisdom of people around the world. They contain many metabolites with therapeutic effects. S. mountain garlic is commonly used for pain therapy in villages near the Himalayas [29]. This work validated that the LAE of S. mountain garlic has an anticoagulant effect *in vivo*. An increased bleeding time was observed with 500 and 1000 mg/kg, which showed significant effects at 1, 2, and 4 weeks, with the strongest effects observed at 2 and 4 weeks (*p* < 0.05). The ASA-treated group demonstrated significant effects at 30 min and 2 weeks. However, from week 8, the pairwise effects began to diminish across all groups; however, the differences among groups remained statistically significant at both weeks 8 and 12.

Similarly, a universal anticoagulant effect (AUC) was shown during the 90 days with the dose of the LAE at 500 mg/kg and 100 mg/kg of ASA. In contrast to ASA, which resulted in duodenal ulcers, acanthosis, and lymphoplasmacytic inflammatory infiltrate, doses below 500 mg showed no histopathological alterations. However, at doses of 1000 mg/kg and 2000 mg/kg, histopathological changes similar to those induced by ASA were observed.

The ED_50_ *in vivo* was 366 ± 2.43 mg/kg. Most work that has been conducted to evaluate the clotting time or bleeding *in vivo* of garlic *Allium sativum* L. has used ethanol extracts, garlic oils, and garlic powder, among others, with doses of 10 mg/kg/d, 50 mg/kg/d, and 1000 μg/mL, respectively, in short periods of 2 to 4 weeks; these show a wide variety of doses and extracts whose results are not conclusive regarding their use in humans [20]. On the other hand, in our work, doses of 250 mg/kg/d and 500 mg/kg/d had no histopathological findings in tissues; results like those found by Bermudez et al., 2015, with the administration of 200 mg/kg of *Allium sativum* L., showed that it has hepatoprotective activity against the toxic action of paracetamol [34].

Nine organosulfate compounds were identified using GC-QToF-MS. Diallyl disulfide (DADS) and diallyl trisulfide (DATS) are the most-studied compounds; evidence has been found to show that these have an inhibitive effect on platelet aggregation [35,36]. On the other hand, allyl methyl trisulfide is documented to have an antiplatelet effect, but this is less documented than it is for DADS and DATS [37]. Diallyl sulfide is a bioactive compound of common garlic, but its anticoagulant activity is not as well documented as it is in the cases of DADS and DATS. On the other hand, a study by Kaur 2022 identified the sulfurous compounds of the garlic S. mountain vs. the common garlic (*Allium sativum* L.); they identified new organosulfate compounds that were not found in *Allium sativum* L.: methanethiol, dimethyl sulfide, propyl mercaptan, 1-allyl-2-isopropyldisuldide, pentadecanoic acid, and Cholesta-4-6-dien-3-ol,(3-beta). In our work, new organosulfur compounds were identified that had not been described for Snow mountain garlic: Thiophene 3,4-dimethyl, Methyl (E)-1-propenyl disulfide, (Z)-1-allyl-2-(prop-1-en-1-yl) disulfan, and Allyl prop-1-enyl disulfide [25].

Novel oral anticoagulants (NOACs) have been in clinical use for several years; however, significant knowledge gaps remain regarding their optimal utilization [38]. This underscores the importance of ongoing research and development of new anticoagulants to enhance patient outcomes and minimize adverse effects. Medicinal plants, such as S. mountain garlic, present an excellent option in this context. For example, NOACs are not indicated for the resolution of intracardiac thrombus; however, there are case reports where they have been used with positive results [38], revealing the need for more studies on these drugs. On the other hand, patients with severe COVID-19 often develop coagulopathies, a major cause of numerous deaths. Treatment for these patients typically involves the use of anticoagulants, which must be promptly discontinued if bleeding occurs [39]. In this context, studying the anticoagulant properties of S. mountain garlic becomes particularly relevant. Traditionally, this type of garlic has been consumed by Himalayan locals for its medicinal benefits [25].

Therefore, the present study examined the *in vivo* anticoagulant effect of S. mountain garlic in a model animal. The anticoagulant effect observed in S. mountain garlic opens new possibilities for its potential use in managing patients with thromboembolic diseases and in the preventive control of such conditions.

In a previous study conducted by our research group [40], we emphasized the importance of personalized dosing based on the specific health condition of each patient and the molecular site of action responsible for the anticoagulant effect. In the case of S. mountain garlic, it remains unclear whether the molecules with anticoagulant properties act synergistically or individually.

Genetic variation should also be taken into account in pharmacological development to ensure safety and efficacy. Additionally, pharmacogenomics plays a crucial role, as genetic variations can affect drug receptors, the proteins involved in drug distribution, and patient responses to the same medication [41].

Therefore, it is important to elucidate each organosulfate compound that was found in LAE to identify its mechanism of action and thus propose individual or synergistic therapeutic doses [42]. Organosulfurate compounds were identified that had not previously been reported in S. mountain garlic. Furthermore, the fibrinolytic effect of this garlic remains unexplored.

## 5. Conclusions

This paper represents the first time that a study of LAE from S. mountain garlic has been carried out to evaluate its anticoagulant effect *in vivo*, scientifically demonstrating its possible usefulness as a treatment for thrombosis in the future. The LAE has been shown to have an anticoagulant effect *in vivo*. The ED_50_ of the LAE *in vivo* was 366 ± 2.43 mg/kg. The toxicity results show that safe use is possible at doses of 250 mg/kg and 500 mg/kg. Similarly, the AUC of the 500 mg/kg/d dose shows an anticoagulant effect like that of 100 mg/kg/d of ASA; however, unlike ASA, it did not show histopathological damage. It is important to establish a mechanism of action that explains the anticoagulant effect observed here. The clinical study represents the next phase of the research to be conducted.

## Figures and Tables

**Table 1:** Kruskal–Wallis and Dunn’s Test for different treatment periods vs. control.

Period	K-W H	K-W *p*-Value ^1^	Treatment	Dunn’s Test ^1,2^	Median	IQR
Time 0	10.897	0.0535	Control	–	213.0	46.5
			ASA	1.0000	208.5	57.5
			250	0.0142 *	306.5	26.5
			500	0.2110	259.0	51.8
			1000	0.8610	198.5	49.2
			2000	0.9402	199.0	102.8
30 min	8.166	0.1473	Control	–	213.0	46.5
			ASA	0.0124 *	296.5	52.2
			250	0.0939	275.0	25.0
			500	0.0403 *	289.0	98.8
			1000	0.1041	281.5	54.0
			2000	0.0187 *	310.0	74.0
1 Week	9.810	0.0808	Control	–	231.5	22.2
			ASA	0.0512	325.0	176.5
			250	0.0214 *	348.5	118.5
			500	0.0093 **	394.0	196.0
			1000	0.0069 **	371.0	45.5
			2000	0.0643	313.5	13.2
2 Weeks	13.223	0.0214 *	Control	–	209.0	25.5
			ASA	0.0315 *	427.5	91.2
			250	0.0229 *	374.0	40.0
			500	0.0027 **	439.5	164.0
			1000	0.3295	275.0	51.8
			2000	0.5484	245.0	20.8
4 Weeks	15.971	0.0069 **	Control	–	228.0	28.2
			ASA	0.5822	266.5	66.2
			250	0.0008 **	548.0	106.5
			500	0.0175 *	450.0	103.8
			1000	0.0187 *	444.0	32.2
			2000	0.2021	330.0	193.0
8 Weeks	17.235	0.0041 **	Control	–	195.0	106.8
			ASA	0.0023 **	386.5	58.0
			250	0.1210	290.5	43.8
			500	0.9601	201.0	45.2
			1000	0.0107 *	375.0	112.5
			2000	0.2452	347.5	106.2
12 Weeks	16.749	0.0050 **	Control	–	340.0	36.2
			ASA	0.2018	422.5	153.8
			250	0.4528	300.0	81.0
			500	0.0936	515.0	41.0
			1000	0.1398	552.0	208.8
			2000	0.1537	233.0	27.2

^1^ *p* < 0.05 (*), *p* < 0.01 (**). ^2^ treatment vs. control.

**Table 2:** Compounds identified using GC-QToF-Ms in the LAE of S. mountain garlic.

Peak	Tr (min)	Metabolite	Formula	CAS
1	11.21	Diallyl sulfide	C6H10S	592-88-1
2	16.58	Thiophene, 3,4-dimethyl	C6H8S	632-15-5
3	18.13	Methyl 2-propenyl disulfide	C6H8S2	217958-0
4	18.56	Methyl (E)-1-propenyl disulfide	C4H8S2	22383-19-9
5	28.84	(Z)-1-Allyl-2-(prop-1-en-1-yl)disulfane	C6H10S2	122156-03-0
6	29.72	Diallyl disulfide	C6H10S2	2179-57-9
7	30.07	Allyl prop-1-enyl disulfide	C6H10S2	122156-02-9
8	35.78	Allyl methyl trisulfide	C4H8S3	34135-85-8
9	46.57	Diallyl trisulfide	C6H10S3	2050-87-5

## Data Availability

The original contributions presented in this study are included in the article. Further inquiries can be directed to the corresponding author.
